# Allometric Growth and Scaling of Body Form of the Spadenose Shark (*Scoliodon laticaudus*)

**DOI:** 10.1002/ece3.70414

**Published:** 2024-10-10

**Authors:** Joel H. Gayford, Ronak Waghe, Phillip C. Sternes, Zoya Tyabji

**Affiliations:** ^1^ Department of Life Sciences Imperial College London London UK; ^2^ College of Science and Engineering James Cook University Townsville Australia; ^3^ Shark Measurements London UK; ^4^ St. Xaviers College Mumbai India; ^5^ Department of Evolution, Ecology and Organismal Biology University of California Riverside California USA; ^6^ Department of Biology Dalhousie University Halifax Canada

**Keywords:** allometry, Elasmobranchii, evolution, isometry, morphology, morphometry

## Abstract

The versatility of the shark body form is suggested to be one of the key factors underlying their evolutionary success and persistence. Nevertheless, sharks exhibit a huge diversity of body forms and morphological adaptations. More subtly, it is increasingly evident that in many species, morphology varies through ontogeny. Multiple competing hypotheses exist explaining both the function of specific morphological structures and the interspecific distribution of these ontogenetic morphological shifts. However, existing studies are restricted to a small number of mostly large‐bodied species. In this study, we report allometric scaling relationships from functionally important morphological structures in the spadenose shark (*Scoliodon laticaudus*). We find that a mosaic of isometric and allometric growth underlies the scaling trends in this species and that cases of allometry are consistent with an ontogenetic shift in diet. Moreover, our results refute suggestions that small‐bodied sharks grow isometrically. Given the small number of existing studies of ontogenetic morphometry in sharks and the life‐history/ecological characteristics of *S. laticaudus*, this study is a valuable contribution to our understanding of the adaptive value of ontogenetic morphological shifts in elasmobranchs.

## Graphical abstract

Multiple hypotheses have been posed to explain differences in ontogenetic scaling amongst shark species. We report both isometric and allometric growth of functionally important structures in the spade nose shark (*Scoliodon laticaudus*). These scaling relationships are consistent with the allometric niche shift hypothesis, but refute the idea that small‐bodied sharks grow isometrically.

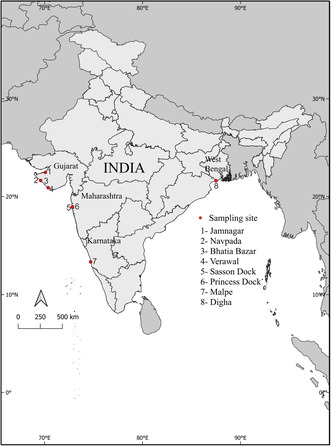

## 1 Introduction

Ontogenetic shifts in morphology have been documented in a wide range of taxa (Hjelm, Persson, and Christensen 2000; Kolarov, Ivanović, and Kalezić 2011; Irschick and Hammerschlag 2015; Patterson et al. 2022). The selective drivers of these morphological shifts vary between systems and can include ontogenetic shifts in diet, predation or habitat usage, trade‐offs with other functionally important traits, or fundamental evolutionary constraints (Pélabon et al. 2014; Voje et al. 2014; Gayford et al. 2023). Studying these shifts in morphology is beneficial as they provide case studies for understanding the process of adaptation and the interplay between selection and constraint, particularly where knowledge of the genetic‐developmental underpinnings of morphology are well understood (Pélabon et al. 2014; Voje et al. 2014). Understanding how morphology and ecological niche change over the course of ontogeny is also important from a management perspective, particularly in the case of taxa threatened with extinction (Bellodi et al. 2023).

Elasmobranchii (sharks and rays) are amongst the most threatened vertebrate clades (Dulvy et al. 2021), and due to their key phylogenetic position are of great importance to our understanding of trait evolution across jawed vertebrate phylogeny (Wilga, Wainwright, and Motta 2000; Cole and Currie 2007; Stein et al. 2018). Recently a number of studies have addressed ontogenetic scaling trends in elasmobranch species: existing studies show that there is substantial variation in the nature and intensity of these ontogenetic morphological shifts not only between species but between different life stages and between the sexes (Summers, Ketcham, and Rowe 2004; Lingham‐Soliar 2005a; Reiss and Bonnan 2010; Scacco, La Mesa, and Vacchi 2010; Irschick and Hammerschlag 2015; Fu et al. 2016; Ahnelt et al. 2020; Sternes and Higham 2022; Bellodi et al. 2023; Gayford, Godfrey, and Whitehead 2023; Gayford et al. 2023; Yun and Watanabe 2023; Gayford, Whitehead, and Jaquemet 2024; Seamone et al. 2024). Several hypotheses have been posed to explain interspecific and intraspecific scaling trends in elasmobranchs: it has been suggested that smaller‐bodied species are likely to grow isometrically, with larger‐bodied species more likely to exhibit allometric shifts in body form as a result of fundamental constraints associated with increased body size (Irschick and Hammerschlag 2015; Ahnelt et al. 2020). Alternatively, the allometric niche shift (ANS) hypothesis suggests that species that undergo ontogenetic shifts in trophic niche or habitat usage are more likely to display allometric growth in aspects of morphology that play a key role in locomotion—such as the caudal, pectoral and dorsal fins (Gayford et al. 2023). Recently, it has also been suggested that allometric growth in these structures may act to conserve, rather than modify hydrodynamic function (Seamone et al. 2024). Unfortunately, existing studies are limited to ~4% of extant species (and in some cases, studies only address ontogenetic scaling in specific structures such as the head or caudal fin), and thus our ability to interpret the adaptive value (or lack thereof) of these ontogenetic morphological shifts is at present limited. Notably, almost all existing studies target large‐bodied species, making it challenging to assess the hypothesis that small‐bodied sharks grow isometrically. For this reason, additional studies are warranted. In particular, species with unusual morphological specializations (i.e., thresher sharks, hammerheads and sawsharks), or particularly large/small body sizes (i.e., lamniforms, pelagic carcharhinids and lantern sharks) should be studied.

The Spadenose shark *Scoliodon laticaudus* is a small‐bodied carcharhiniform shark distributed throughout the shallow coastal and estuarine waters of the Indian Ocean (Ebert, Dando, and Fowler 2021; Lim et al. 2022; Sukumaran et al. 2023). Whilst pelagic prey items have been found in the stomachs of *S. laticaudus* individuals, it is thought to primarily be a demersal/benthopelagic species, inhabiting sandy and rocky bottoms between 50 and 80 m in depth (Wai et al. 2012; Lim, Then, and Loh 2023). Typical prey species include teleost fishes, crustaceans, cephalopods and polychaete worms (Wai et al. 2012; Lim, Then, and Loh 2023). Adults and juveniles are known to co‐occur in estuarine environments and there is no evidence for spatial segregation or ontogenetic shifts in habitat usage between size classes (Wai et al. 2012; Bhavan et al. 2023). Despite this, multiple studies have recovered evidence of marked ontogenetic dietary shifts in *S. laticaudus*, with larger individuals feeding on more agile teleost prey, whilst smaller individuals predominantly target slow‐moving crustaceans (Wai et al. 2012; Lim, Then, and Loh 2023). This may be due to differential energetic requirements between size classes, or an adaptive mechanism of reducing competition between adults and juveniles (Lim, Then, and Loh 2023). There is no direct evidence of ecological differences between the sexes of *S. laticaudus*, although females do mature at a notably larger size than males (Ebert, Dando, and Fowler 2021). The small size of *S. laticaudus* (reaching a maximum total length of 74 cm, see Ebert, Dando, and Fowler 2021) makes it an ideal species through which to study scaling and allometry. Such shifts have only previously been studied in one species smaller than *S. laticaudus*—*Etmopterus spinax* (Bellodi et al. 2023). Moreover, as *S. laticaudus* exhibits ontogenetic shifts in diet but not habitat usage (Lim, Then, and Loh 2023), it may provide insight into the extent to which dietary shifts alone are sufficient to select for ontogenetic shifts in morphology.

In this study, we utilise traditional linear morphometrics to investigate ontogenetic shifts in morphology and body form from a dataset of *S. laticaudus* individuals landed in commercial and artisanal fisheries, including both juveniles and adults. Assuming the hypothesis of small shark species growing isometrically, we would not expect to see allometric growth in functionally important structures such as the caudal, dorsal or pectoral fins of *S. laticaudus*. However, if allometric scaling relationships are dictated primarily by changes in habitat usage and trophic ecology rather than body size (as predicted by the ANS hypothesis), we might expect to observe allometric growth in these structures, given the trophic niche shift seen in *S. laticaudus* (Lim, Then, and Loh 2023).

## 2 Materials and Methods

**FIGURE 1:**
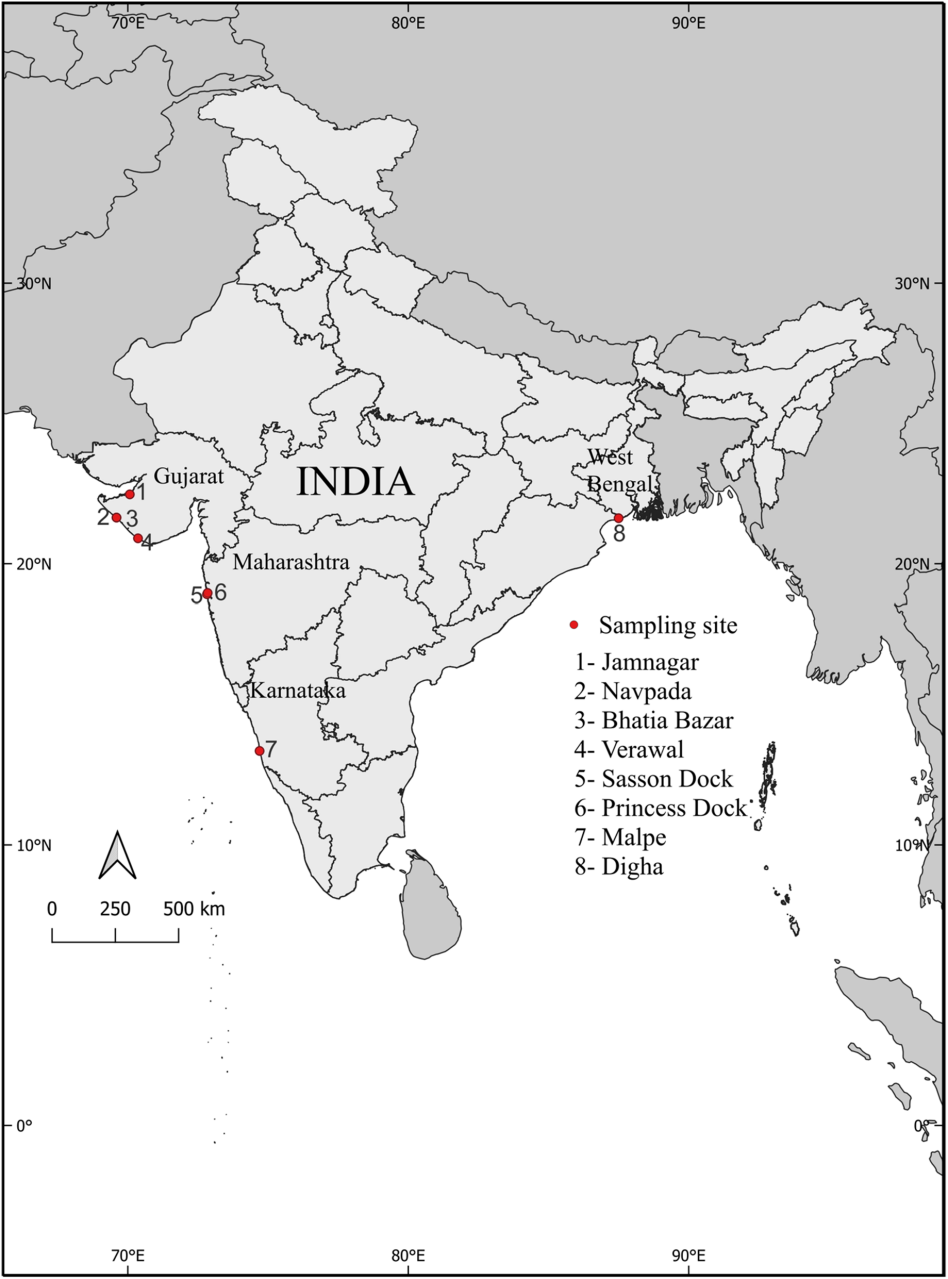
Map of India showing all the fish landing and auction sites where data were collected, demarcated by red dots.

**FIGURE 2:**
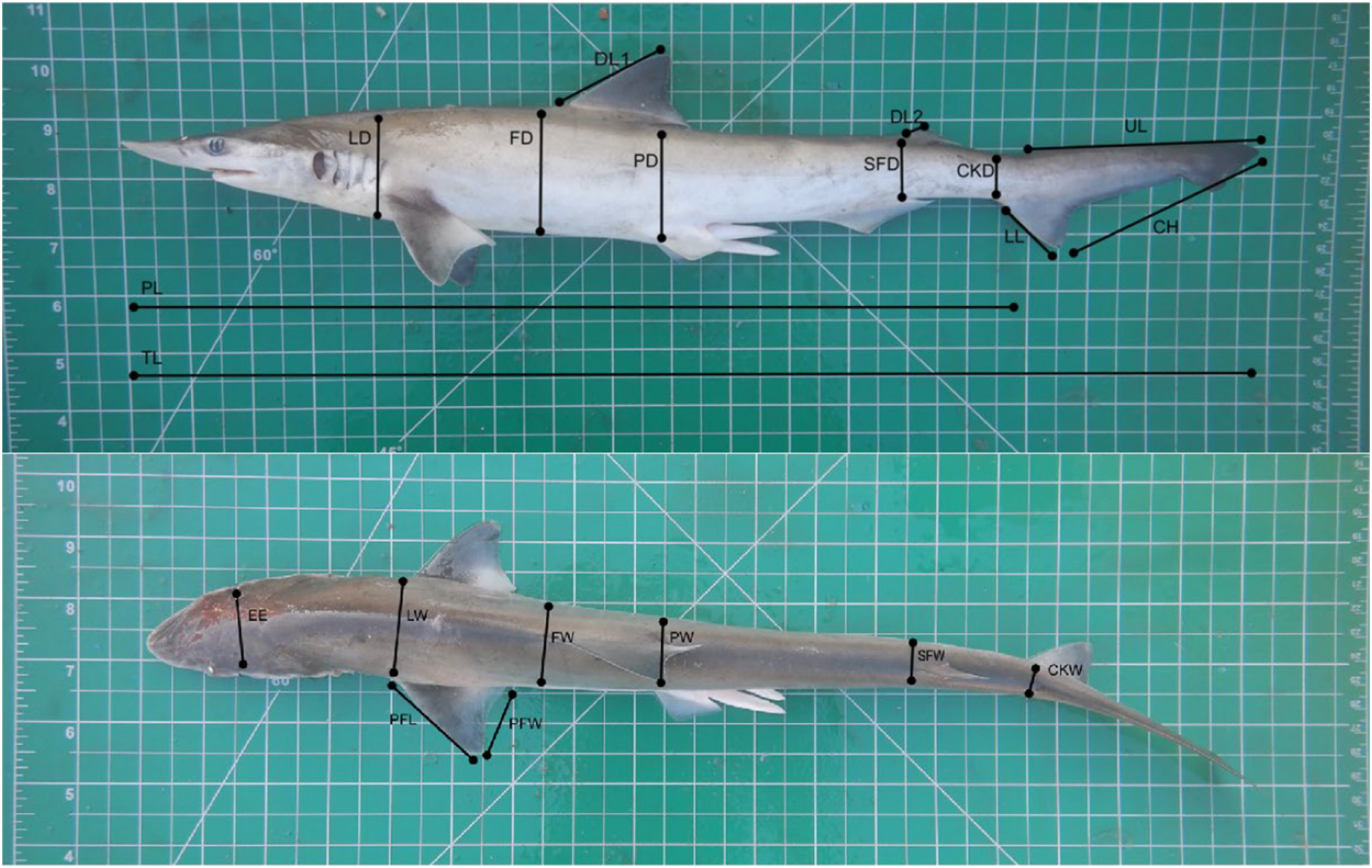
Lateral and dorsal images of a *Scoliodon laticaudus* individual used to extract morphological measurement data. Note that measurement labels are not exact and merely illustrate the region of the body in which each measurement was taken. For a detailed morphological description of how measurements were extracted, see Table 1.

Photographic data were opportunistically collected from various fish landing sites and auctioneering markets in India (Figure 1) between October 2022 and April 2023. At these sites, sharks were carefully placed on an A3 size architect cutting mat, after which a photo was taken at an angle of 90° above the individual to avoid any errors that may have been caused due to the angle of photographs (Figure 2). A photo of the lateral and dorsal view of the individuals was taken, along with individual photos of body appendages including the caudal and pectoral fins. This allowed us to extract a large number of morphological measurements (Table 1), which would not have been possible at fish landing sites due to the fast‐paced nature of catch processing. Morphological measurements were selected in line with previous studies (Irschick et al. 2017; Gayford, Godfrey, and Whitehead 2023), and included various measurements of girth, and measurements of the pectoral, dorsal and caudal appendages (Table 1).

**TABLE 1:** Morphological measurements extracted from photographic data collected in this study.

Measurement	Abbreviation	Morphological description
Total length	TL	Distance from the tip of the snout to the dorsal tip of the caudal fin
Precaudal length	PL	Distance from the tip of the snout to the precaudal pit
Lateral width	LW	Linear distance across the dorsal body surface, measured between the anterior insertion points of the pectoral fins
Frontal width	FW	Linear distance across the dorsal body surface at the anterior insertion point of the first dorsal fin, measured between the horizontal plane of one pectoral fin and the horizontal plane of the other pectoral fin
Proximal width	PW	Linear distance across the dorsal body surface at the posterior insertion point of the first dorsal fin, measured between the horizontal plane of one pectoral fin and the horizontal plane of the other pectoral fin
Second frontal width	FW2	Linear distance across the dorsal body surface at the anterior insertion point of the second dorsal fin, measured between the horizontal plane of one pectoral fin and the horizontal plane of the other pectoral fin
Caudal keel width	CKW	Linear distance across the origin of the caudal fin
Lateral depth	LD	Body depth measured at the anterior insertion point of the pectoral fins
Frontal depth	FD	Body depth at the anterior insertion point of the first dorsal fin
Proximal depth	PD	Body depth at the posterior insertion point of the first dorsal fin
Second frontal depth	SFD	Body depth at the anterior insertion point of the second dorsal fin
Caudal keel depth	CKD	Body depth at the origin of the caudal fin
First dorsal fin length	DL1	Distance from the anterior insertion point of the first dorsal fin to the upper tip of the first dorsal fin
Second dorsal fin length	DL2	Distance from the anterior insertion point of the second dorsal fin to the upper tip of the second dorsal fin
Pectoral fin length	PF	Distance from the anterior insertion point of the pectoral fin to the fully extended tip of the same pectoral fin
Pectoral fin width	PFW	Distance from the anterior insertion point of the pectoral fin to the posterior insertion point of the same pectoral fin
Upper caudal lobe	UL	Distance from the dorsal insertion point of the caudal fin to the dorsal tip of the caudal fin
Lower caudal Lobe	LL	Distance from the ventral insertion point of the caudal fin to the ventral tip of the caudal fin
Caudal height	CH	Distance between the dorsal tip and ventral tip of the caudal fin
Eye to eye distance	EE	Distance between the midpoints of the left and right eyes across the dorsal body surface

Measurements were extracted from the photos using ImageJ, a Java‐based image‐processing program developed at the National Institutes of Health Laboratory for Optical and Computational Instrumentation (LOCI, University of Wisconsin). The photographs of the sampled sharks were run through the program where a scale was set denoting a known distance.

### 2.1 Data Analysis

Prior to statistical analyses, the data were log_10_ transformed in accordance with previous studies (Irschick and Hammerschlag 2015; Sternes and Higham 2022). To determine whether different morphological structures exhibit isometric or allometric growth, we performed linear regression analyses between precaudal length (PL) and each of the measurements not explicitly related to body length (Table 1) using the *R* package *ggplot2* (Wickham, Chang, and Wickham 2016; R Core Team 2023) and following the approach of Gayford et al. (2023). Where isometric growth is observed the scaling coefficient is not expected to differ significantly from 1 for linear measurements, and if any such difference is observed this is indicative of allometric growth (Sternes and Higham 2022). Data were not stratified by life stage or sex due to the presence of missing values in the data where specific measurements could not be taken due to logistical constraints during sampling. The full dataset used in this study can be found in the supporting information associated with this article (Table S1).

## 3 Results

A total of 129 sharks were measured, of which 70 individuals were female and 59 were male. The males ranged from the smallest total length of 25.0 cm to the largest of 53.7 cm (meaning all but the smallest males were sexually mature). For females, the individuals ranged from a smallest total length of 25.5 cm, to a largest of 58.7 cm. Consequently, our dataset includes both juveniles and sexually mature adults according to the most recently published size estimates for the species (Ebert, Dando, and Fowler 2021). Whilst both ‘extremes’ of neonatal individuals and those nearing the maximum‐recorded size of the species are missing, our dataset includes a large number of juveniles and adults, covering the range of ontogenetic stages included in existing studies of spatial and trophic ecology (Lim, Then, and Loh 2023), and a comparable size range to other studies of ontogenetic scaling in sharks.

**FIGURE 3:**
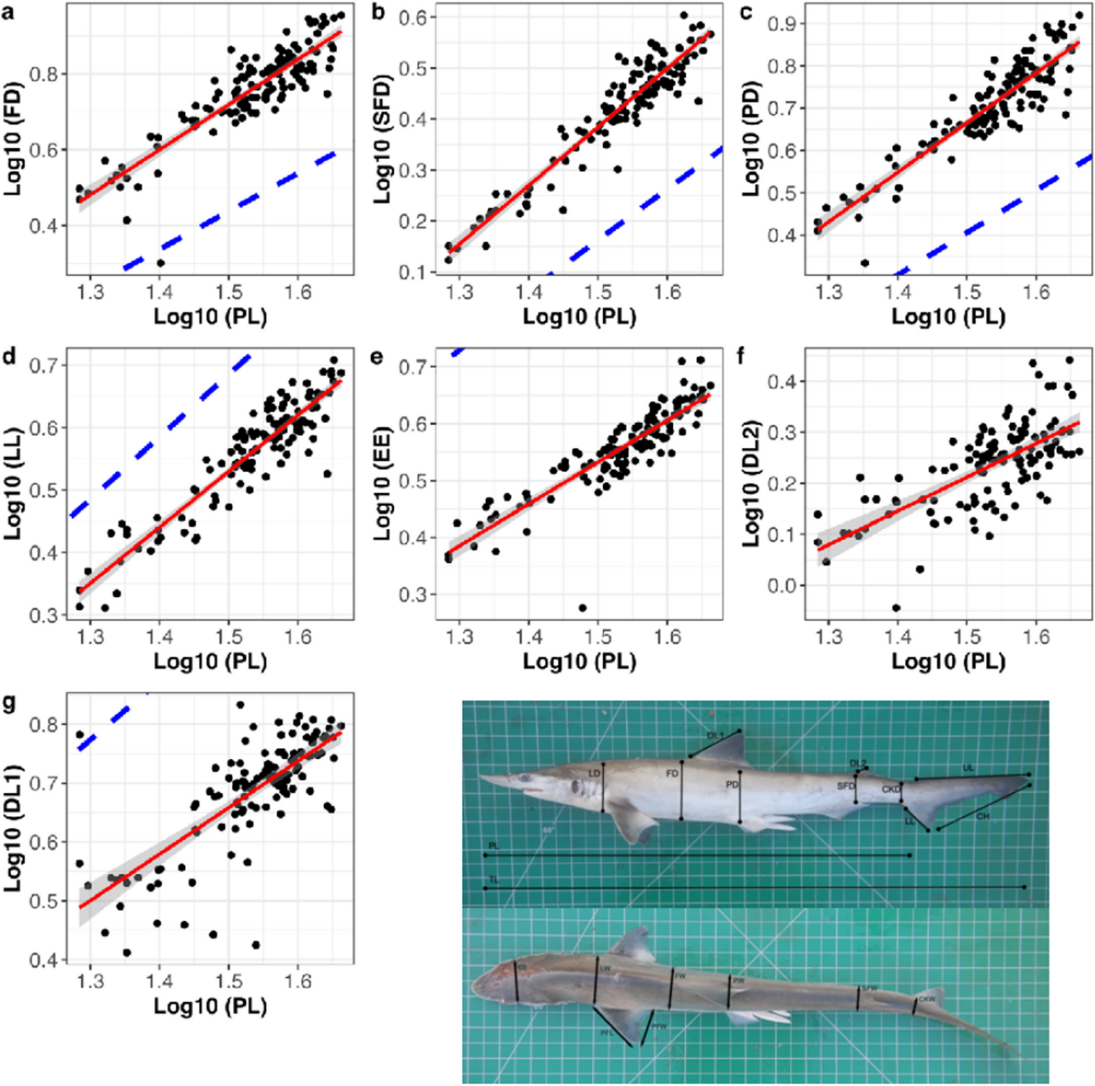
Linear regression plots displaying significant scaling relationships (allometric growth) from Table 2. Specifically, plots display the relationship between precaudal length (PL) and the following measurements: FD (a), SFD (b), PD (c), LL (d), EE (e), DL2 (f), and DL1 (g). All data are log_10_ transformed and the dark grey area represents the 95% confidence interval for the scaling coefficient. Blue dashed lines demonstrate isometric growth. An inset showing the anatomical location of each measurement is included for ease of interpretation.

Regression of 18 linear measurements against precaudal length (PL) revealed seven cases of allometric growth and 11 cases of isometric growth (Figure 3; Table 2). $R^{2}$ varied between 0.47 (Second dorsal fin length; Table 2) to 0.88 (Second frontal depth; Table 2), but in most cases was between 0.70 and 0.85. Of the seven cases of allometric growth, three showed positive allometry with scaling coefficients ranging between 1.15 and 1.19 (Frontal depth, Proximal depth, and Second frontal depth; Figure 3; Table 2) and four showed negative allometry with scaling coefficients ranging between 0.66 and 0.90 (eye to eye distance, second dorsal fin length, lower caudal lobe and first dorsal fin length; Figure 3; Table 2).

**TABLE 2:** Linear regression results for each linear measurement, with significant results (*p* < 0.05) highlighted in bold.

Character	Coefficient	Std. error	*t* value	*p*	Residual std. error	$R^{2}$	Adj. $R^{2}$	*F* value	*N*
LW	0.91308	0.04719	1.842	0.0679	0.04601	0.7543	0.7522	374.5	122
FW	1.10588	0.07349	1.441	0.153	0.06242	0.7247	0.7215	226.4	86
PW	1.1860	0.1161	1.602	0.118	0.07652	0.7381	0.7311	104.3	37
SFW	1.0929	0.1799	0.516	0.61431	0.06102	0.7395	0.7195	36.91	13
CKW	1.1030	0.0749	1.376	0.176	0.04287	0.8313	0.8275	216.9	44
LD	1.02958	0.05134	0.576	0.566	0.04476	0.7658	0.7639	402.2	123
**FD**	**1.18725**	**0.05598**	**3.345**	**0.0011**	**0.05483**	**0.7798**	**0.7781**	**449.7**	**127**
**PD**	**1.17325**	**0.04959**	**3.494**	**6.61e‐04**	**0.04753**	**0.8187**	**0.8172**	**559.8**	**124**
**SFD**	**1.14798**	**0.03770**	**3.925**	**1.43e‐04**	**0.03656**	**0.8829**	**0.8819**	**927.1**	**123**
CKD	1.03533	0.04557	0.775	0.44	0.04372	0.8063	0.8048	516.2	124
**DL1**	**0.78991**	**0.06343**	**3.312**	**1.21e 03**	**0.06198**	**0.5537**	**0.5501**	**155.1**	**125**
**DL2**	**0.66318**	**0.06388**	**5.273**	**6.03e‐07**	**0.06083**	**0.4732**	**0.4688**	**107.8**	**120**
PFL	1.05279	0.04005	1.318	0.19	0.03785	0.8552	0.854	691.1	117
PFW	1.0101	0.1323	0.077	0.939	0.04066	05756	0.5658	58.33	43
UL	0.97551	0.03385	0.723	0.471	0.03311	0.8692	0.8681	830.4	125
**LL**	**0.89652**	**0.03413**	**3.032**	**2.96e‐03**	**0.03338**	**0.8466**	**0.8454**	**689.8**	**125**
CH	0.97586	0.09288	0.26	0.7959	0.03122	0.6674	0.6614	110.4	55
**EE**	**0.73498**	**0.03627**	**7.306**	**2.85e‐11**	**0.03531**	**0.7666**	**0.7647**	**410.5**	**125**

*Note:* Significance implies that the scaling coefficient of the measurement in question against precaudal length (PL) differs significantly from 1, consistent with allometric growth. *N* refers to the number of individuals from which this measurement was gathered (the sample size).

## 4 Discussion

Ontogenetic shifts in shark caudal fin morphometry are relatively well studied from an ecomorphological perspective (Lingham‐Soliar 2005a; Fu et al. 2016; Ahnelt et al. 2020; Sternes and Higham 2022; Bellodi et al. 2023; Gayford, Godfrey, and Whitehead 2023; Gayford et al. 2023; Yun and Watanabe 2023). Changes to the relative size and geometry of the caudal fin can have significant implications for locomotor efficiency and swimming performance (Lauder 2000; Wilga and Lauder 2002; Aalbers, Bernal, and Sepulveda 2010; Iliou et al. 2023; Sumikawa et al. 2023). Sharks are characterised by a heterocercal caudal fin, where the upper lobe is longer than the lower lobe, although exceptions do exist (Thomson 1976; Lauder 2000; Sternes and Shimada 2020; Giammona 2021). In several species, particularly those that exhibit ontogenetic shifts in habitat usage and/or trophic ecology, the caudal fin appears to become less heterocercal through ontogeny (Lingham‐Soliar 2005a; Fu et al. 2016; Ahnelt et al. 2020; Sternes and Higham 2022; Gayford et al. 2023). This reflects a trade‐off between the agility and manoeuvrability afforded by a relatively heterocercal caudal fin, and the locomotor efficiency afforded by a relatively homocercal caudal fin, which counteracts the decreasing lift/drag ratio in larger‐bodied individuals (Gayford et al. 2023) and facilitates efficient, high‐speed cruising (Iliou et al. 2023; Seamone et al. 2024).

Contrary to this, we find that in *S. laticaudus* the upper caudal lobe exhibits isometric growth, and the lower caudal lobe exhibits negative allometric growth (Figure 3f; Table 2), such that the caudal fin becomes more heterocercal through ontogeny. As *S. laticaudus* is not known to exhibit any migratory behaviour and is primarily restricted to coastal and estuarine waters (Devadoss 1989; Sukumaran et al. 2023), a relatively homocercal caudal fin would likely provide minimal benefit. This is particularly true in the benthic environments occupied by *S. laticaudus*, where high‐speed cruising behaviour is unlikely to occur. Indeed, extremely heterocercal caudal fins are not uncommon amongst benthic shark species (Thomson 1976; Compagno 1990; Sternes and Shimada 2020). In the absence of any known differences in habitat usage between adults and juveniles, this ontogenetic trend towards increased asymmetry in the caudal fin is consistent with ontogenetic shifts in trophic ecology observed in *S. laticaudus*. Whilst juveniles feed predominantly on stationary or slow‐moving prey such as crustaceans, adults predominantly target more mobile, agile prey such as teleost fishes (Wai et al. 2012; Lim, Then, and Loh 2023). Thus, in larger‐bodied individuals a more heterocercal caudal fin, providing greater manoeuvrability and turn speed could be favoured (Lauder 2000). It is worth noting that how this ontogenetic change to caudal geometry would influence feeding performance in the pelagic realm remains unknown—and *S. laticaudus* is known to feed upon pelagic prey species at times (Wai et al. 2012; Lim, Then, and Loh 2023).

Several different adaptive hypotheses have been posed to explain interspecific and intraspecific differences in girth scaling in elasmobranchs, including positive liver allometry (Lingham‐Soliar 2005a, 2005b; Iosilevskii and Papastamatiou 2016; Gleiss, Potvin, and Goldbogen 2017), ontogenetic shifts in energy storage (Gallagher et al. 2014; Irschick and Hammerschlag 2014), and hydrodynamic performance, although functional studies are yet to unravel the exact nature of this relationship (Musick 1990; Iosilevskii and Papastamatiou 2016; Sternes and Higham 2022). It appears that the extent of negative girth allometry observed across ontogeny correlates positively with the extent of migratory behaviour (Gayford, Godfrey, and Whitehead 2023). Our results are consistent with this idea, as *S. laticaudus*, a species not thought to exhibit any migratory behaviour, demonstrates positive allometric growth across much of the trunk (Figure 3a–c; Table 2). Such positive allometry might not be expected in migratory taxa as it may incur significant energetic costs and reduce efficiency of locomotion over large distances (Musick 1990; Iosilevskii and Papastamatiou 2016; Gayford, Godfrey, and Whitehead 2023).

Positive allometry is not observed in all girth measures of *S. laticaudus*, as the head appears to become narrower through ontogeny (Figure 3d; Table 2) whilst the precaudal region of the trunk exhibits isometric growth (Table 2). In the case of negative allometry in the head, it is plausible that this represents an adaptation for drag‐reduction, however in light of the previously described positive allometry in the trunk this seems unlikely. Given the known ontogenetic dietary shift in *S. laticaudus*, adults and juveniles are likely to require different arrangements of musculature to successfully acquire, handle and process prey. Specifically, juveniles have a somewhat durophagous diet compared to adults, that feed predominantly on teleost fishes (Wai et al. 2012; Lim, Then, and Loh 2023). Both the external morphology and mechanical properties of the jaws and head are known to shift through ontogeny in a number of species—including both durophagous and piscivorous taxa (Summers, Ketcham, and Rowe 2004; Huber, Weggelaar, and Motta 2006; Lowry, Motta, and Hueter 2007; Lowry and Motta 2007; Kolmann and Huber 2009; Fu et al. 2016). Typically bite force—through changes to the size of the jaws and jaw adductor muscles—increases with positive allometry through ontogeny (Huber, Weggelaar, and Motta 2006; Fu et al. 2016). In the bull shark, this positive allometry is thought to be associated with a lateral broadening of the head and hypertrophy of the jaw adductor muscles (Habegger et al. 2012; Gayford, Whitehead, and Jaquemet 2024), providing a ‘performance increase’ that allows smaller individuals to increase the breadth of their trophic niche. If laterally broad heads and jaws are generally associated with increased bite force, then the progressive ontogenetic narrowing of the head relative to body size in *S. laticaudus* would suggest that smaller individuals possess greater bite force for their size relative to larger adults. Whilst this may seem unusual, hard‐shelled benthic prey items require relatively high bite forces to handle and process (Kolmann and Huber 2009). Thus, a broader head—presumably accompanied by jaw musculature adapted for the consumption of such prey—would enable juveniles to forage successfully on hard‐shelled prey items.

Both the first and the second dorsal fins of *S. laticaudus* exhibit negative allometric growth (Figure 3e; Table 2). In the case of the first dorsal fin this result is consistent with a broad trend observed in other carcharhiniform sharks, where the dorsal fin appears to become taller and narrow through ontogeny as a result of negative allometry in one or more measurements (Sternes and Higham 2022; Gayford, Godfrey, and Whitehead 2023; Gayford et al. 2023; Gayford, Whitehead, and Jaquemet 2024). Ontogenetic morphometry of the second dorsal fin has only explicitly been studied in one other species (the bull shark), recovering the same result of negative allometry (Gayford, Whitehead, and Jaquemet 2024). The dorsal fins are thought to provide stabilising or thrust generating functions depending on the species in question (Lingham‐Soliar 2005b; Maia and Wilga 2013; Maia, Lauder, and Wilga 2017), and near identical scaling trends in the first and second dorsal fins of the bull shark have led to speculation that both structures may perform similar functions (Gayford, Whitehead, and Jaquemet 2024). However, in the absence of further functional studies, the underlying drivers of dorsal fin allometry remain unknown.

The elasmobranch pectoral fin is another structure with multiple hypothesised functions, such as initiating turning manoeuvres, maintenance of trim and facilitation of depth changes are amongst the most general and best‐supported hypotheses (Fish and Shannahan 2000; Wilga and Lauder 2001; Hoffmann and Porter 2019). In *S. laticaudus* we found that pectoral fins grow isometrically (Table 2), similarly to another benthopelagic shark species *Mustelus henlei* (Gayford, Godfrey, and Whitehead 2023). Considering the increased importance of manoeuvrability and agility of larger‐bodied individuals to facilitate the capture of teleost prey we might expect some form of allometric growth in the pectoral fins. However, the clear caudal allometry present in this species (Figure 3f; Table 2) combined with an absence of ontogenetic shifts in habitat usage may compensate for a lack of manoeuvrability conveyed by the pectoral fins of adults relative to juveniles.

In addition to providing insight into the potential function and ecomorphology of shark fins, and the ecology of *S. laticaudus*, our results have important implications for our understanding of the evolutionary causes and consequences of allometric growth. Crucially, the apparent allometric growth trajectories of the dorsal and caudal fins (Figure 3; Table 2), combined with a maximum total length of 74 cm (Ebert, Dando, and Fowler 2021), strongly suggest that allometric growth in shark body form is not restricted to larger‐bodied species. Of course, allometric growth in some aspects of *S. laticaudus* morphology does not rule out the prospect of any relationship between body size and body form allometry across shark diversity, however it does contradict previous speculation that small‐bodied sharks grow isometrically (Irschick and Hammerschlag 2015; Ahnelt et al. 2020). We also cannot rule out the conservation of hydrodynamic function hypothesis (i.e., that the observed allometric growth acts to maintain hydrodynamic function), as this would require additional information regarding the locomotor behaviour of *S. laticaudus*, and the collation of further hydrodynamically relevant data such as tissue density and fin aspect ratios (e.g., Seamone et al. 2024). However, our results are consistent with the suggestion that allometric growth acts to optimise performance in species that exhibit shifts in trophic and/or spatial ecology through ontogeny (the ANS hypothesis). Specifically, allometric trajectories observed in the trunk, head and caudal fin (Table 2) correspond to a shift from a comparatively sedentary and durophagous lifestyle in juveniles, to a more active, piscivorous diet in adults (Lim, Then, and Loh 2023). In light of this niche shift, the observed allometric growth trajectories of the trunk, head and caudal fin (Figure 3; Table 2) may have evolved to provide greater manoeuvrability through ontogeny, and relatively high bite force in juveniles. Additional functional studies are needed to determine exactly how these morphological shifts may influence hydrodynamic forces across the body, but at present our results suggest that niche shift‐induced natural selection likely underlies the scaling of body form in *S. laticaudus*.

## 5 Conclusions

We have found that *S. laticaudus* exhibits a combination of allometric and isometric growth in functionally important aspects of morphology, as observed in other shark species (Irschick and Hammerschlag 2015; Bellodi et al. 2023; Gayford et al. 2023). *S. laticaudus* is a demersal species not known to exhibit ontogenetic shifts in habitat usage, however observed cases of allometry are consistent with trophic differences between adults and juveniles or fundamental constraints relating to body size. Even though we were unable to include the extremes of *S. laticaudus*'s size range, our dataset was sufficient to identify allometric growth in body form in this small‐bodied species, refuting previous suggestions that small‐bodied species grow isometrically. However, numerous questions remain unanswered and the function of several morphological structures remains entirely unknown. In order to better understand the adaptive value of ontogenetic shifts in elasmobranch morphology additional studies (functional, comparative and evo‐devo) will be required.

## Author Contributions

**Joel H. Gayford:** conceptualization (lead), formal analysis (lead), methodology (lead), writing – original draft (lead), writing – review and editing (lead). **Ronak Waghe:** data curation (equal), writing – original draft (supporting), writing – review and editing (supporting). **Phillip C. Sternes:** writing – original draft (supporting), writing – review and editing (supporting). **Zoya Tyabji:** conceptualization (supporting), data curation (equal), funding acquisition (lead), writing – original draft (supporting), writing – review and editing (supporting).

## Conflicts of Interest

The authors declare no conflicts of interest.

## Supporting information


**Table S1.** Full raw data including all measurements, sex and sampling locations.

## Data Availability

The data underlying this article are available in the article and in its Table S1.
