# Supplementary material for: Allometric Growth and Scaling of Body Form of the Spadenose Shark (Scoliodon laticaudus)
Source: Ecol Evol. 2024 Oct 10;14(10):e70414. doi: 10.1002/ece3.70414 (PMC11467038; doi:10.1002/ece3.70414)
Supplement: Supplementary file 1 — Table S1. Full raw data including all measurements, sex and sampling locations. [file ECE3-14-e70414-s001.pdf]

Supplementary materials for ‘Ontogenetic shifts in the body form of the spadenose shark (Scoliodon laticaudus)’

Table S1: full raw data including all measurements, sex and sampling location.

Location	Sex	TL	PL	LD	LW	FD	SFD	FW	SFW	PW	PD	EYD	PFL	PFW	DL2	UL	LL	CH	CKW	CKD	DL1
Karnataka	M	47.922	37.622	4.956	4.212	5.408	2.667	3.636	1.92	3.017	4.494	3.4	4.522		1.757	9.821	3.486		1.497	2.072	5.05
Gujarat	M	47.455	36.053	4.825	4.418	5.945	3.271				5.421	3.935	5.01		2.151	11.24	4.345			2.217	5.400
Gujarat	M	41.908	33.289	4.562	3.834	6.144	2.48	3.88				3.259	3.924		1.791	8.8	3.536		1.307	1.855	5.037
Gujarat	F	35.041	27.999	4.221	3.708	4.755	2.4	2.841			4.097	3.346			1.39	7.179	2.798		1.232	1.634	3.397
Gujarat	F	45.809	35.788	4.677	3.784	5.564	2.553				5	3.908	4.56		1.81	9.895	3.321			2.082	5.268
Gujarat	F	35.855	28.424	3.888	3.328	4.647	2.077	3.206			4.193	3.347	3.722		1.318	7.502	2.652		1.137	1.567	4.134
Gujarat	M	41.178	33.176	4.629	3.941	5.436	2.596	3.515		2.49	4.65	3.588	4.696		1.666	8.551	3.457		1.379	1.876	4.512
Gujarat	M	48.364	37.769	6.005	3.892	6.008	2.847	3.936	1.953	3.06	5.173	3.845	4.601		1.758	10.619	4.073		1.537	2.14	5.191
Gujarat	M	50.334	38.987	4.512	3.891	5.829	3.16				5.405	3.534	4.925		1.786	11.52	4.098			2.147	5.475
Gujarat	M	51.499	40.403	4.499	3.912	5.999	2.957	3.781		3.173	4.994	3.531	4.796		1.501	10.667	4.089			2.092	5.145
Gujarat	M	45.99	35.79	3.903	3.585	5.401	2.811				5.007	3.422	4.621		1.36	9.905	3.826			1.967	4.565
Gujarat	M	44.069	34.072	3.923	3.383	4.981	2.509	2.982		2.63	4.425	3.221	4.184		1.248	9.856	3.917			1.93	4.892
Gujarat	M	42.314	32.484	3.711		5.478	2.637				4.88	3.58	5.864		1.284	9.86	3.839				5.018
Gujarat	F	54.797	42.361	6.204	4.8944	7.197	3.258				6.431	4.401	6.055		2.097	10.727	4.293			2.343	6.120
Gujarat	F	53.569	41.039	5.584	4.451	6.437	3.208	4.117			5.846	4.348			1.934	12.565	4.687			2.446	5.992
Gujarat	M	51.998	41.89	5.254	4.37	6.414	3.065				5.788	3.851	5.83		1.864	10.187	3.822		1.554	2.341	6.425
Gujarat	F	49.932	36.801	4.688	4.177	6.397	2.887	4.255	1.6		5.844	3.676	4.759		1.929	10.124	4.029			2.142	5.020
Gujarat	M	47.689	37.1	4.871	3.991	6.474	2.831				6.093	3.636	4.711		1.431	10.556	4.036			2.162	4.738
Gujarat	M	50.583	39.662	5.553	4.332	6.554	2.982				5.396	3.758	4.675		1.95	10.807	3.939		1.45	2.262	6.350
Gujarat	M	51.024	40.833	4.866	4.471	5.78	3.077				5.263	4.127	4.885		1.98	11.071	4.057		1.62	2.272	6.244
Gujarat	F	36.903	29.368	4.15	3.757	5.126	2.207				4.397	3.548	3.737		1.679	8.292	3.534			1.839	
Gujarat		44.201	34.698	4.621	3.198	5.464	2.513				4.865	3.399	3.852		1.763	9.343	3.658		1.323	1.883	
Gujarat	F	35.615	28.315	3.813	3.284	4.755	2.093	3.199			4.006	3.329	3.804		1.322	7.439	2.628		1.267	1.573	4.182
Gujarat	M	42.417	33.794	4.708	3.736	6.319	2.001	3.914			5.368	3.395	4.366		1.8	8.598	3.429		1.397	1.833	4.943

Gujarat	M	42.087	33.423	4.638	3.904	5.421	2.553	3.492			4.59	3.592	4.758	1.665	8.522	3.512		1.88	4.921
Gujarat	F	34.377	27.047	4.251	3.884	4.919	1.844				3.89	2.933		1.075	7.204	2.853	1.069	1.536	3.600
Gujarat	M	38.764	30.051	3.808	2.158	4.424	2.016	1.972		1.283	3.787	1.889	2.461		8.762	2.977	1.092	1.591	2.769
Gujarat	F	43.534	33.054	3.967	3.436	4.825	2.499	3.128			4.375	3.505	3.633	1.959	10.481	3.925		1.867	4.476
Gujarat	M	47.686	36.576	4.602	3.7	5.758	2.756	3.183		3.067	4.617	3.593	5.136	1.952	11.106	4.281	1.48	2.044	5.341
Gujarat	M	46.23	36.362	5.417	4.231	6.552	3.08	4.141			5.971	3.666	4.843	2.221	9.926	4.417		2.242	5.170
Gujarat	M	49.563	37.791	4.712	3.857	5.527	3.017	3.318		3.016	5.002	3.639	4.802	2.01	11.53	4.282		2.174	5.646
Gujarat	M	47.887	36.248	4.56	4.049	6.328	3.244				5.648	3.95	4.537	2.202	11.313	4.113	1.559	2.134	5.112
Gujarat	M	31.33	24.398	3.351	2.946	4.309	1.638	2.589			3.819	2.849	3.028	1.378	6.848	2.527		1.335	3.331
Gujarat	M	31.865	24.978	2.918	2.885	3.445	1.694	2.297		2.25	3.059	2.569	3.082	0.903	6.784	2.615		1.318	3.382
Gujarat		45.419	34.624	3.852	3.623	4.971	2.709	3.548	2.049	3.34	4.746	3.512	4.142	1.853	10.7	3.879	1.453	1.985	2.659
Gujarat	F	25.537	19.801		2.432	3.059	1.4				2.912	2.66	2.499	1.11	5.452	2.342		1.112	3.353
Gujarat	M	27.322	21.375		2.443	3.29	1.6	2.104		1.816	3	2.637	2.535	1.262	5.42	2.696		1.204	3.458
Gujarat	M	24.988	19.248	2.848	2.434	3.14	1.327	2.259		1.801	2.571	2.338	2.233	1.376	5.624	2.056	0.751	1.056	3.659
Gujarat	M	29.258	22.524	2.707	2.433	3.349	1.79	2.328	1.225	1.781	3.054	2.372		1.288	6.673	2.734		1.332	2.583
Gujarat		28.623	22.048	2.65	2.6588	3.171	1.619				2.762	2.91	2.638	1.248	6.563	2.431		1.252	3.094
Gujarat		30.306	23.392	2.994	2.826	3.17	1.791	2.479			3.226	2.958	3.123	1.474	6.855	2.547		1.404	3.462
Gujarat	F	31.17	25.015	3.159	2.809	4.281	1.782	2.456	1.303	2.061	4.033	2.955	3.275	1.455	6.879	2.727	1.008	1.48	3.572
Gujarat	M	31.461	24.926	2.946	3.117	4.059	1.713	2.84	1.367	2.338	3.654	3.002	3.225	1.467	6.845	2.853	1.047	1.397	2.896
Gujarat	F	28.146	21.792	3.144	2.771	3.41	1.414	2.279		2.204		2.837	2.553		6.245	2.158	0.857		3.433
Gujarat	F	29.32	22.16		3.059	3.579	1.65	2.589			3.261	2.706	2.725	1.626	6.931	2.791		1.243	3.463
Gujarat	F	29.145	22.524	2.863	2.697	2.594	1.66	2.398			2.156	2.751	2.866	1.473	6.521	2.683	0.936	1.288	3.393
Gujarat	M	31.966	25.208	3.064	2.906	2.001	1.838	2.599			3.243	2.887	3.156	1.622	6.481	2.653	0.993	1.405	3.581
Gujarat	M	34.294	27.286	3.995	3.396	4.78	2.081	3.19			4.129	3.289	3.544	1.471	6.811	2.702		1.532	2.878
Gujarat	M	26.461	20.955	2.986	2.42	3.72	1.537	2.57			3.083	2.423	2.72	1.266	5.51	2.048		1.22	2.789
Gujarat	F	25.506	19.257		2.25	2.941	1.415	2.037		1.898	2.695	2.299	2.392	1.214	6.199	2.186		1.069	6.055
Gujarat	M	53.697	41.478	5.492	4.302	6.711	3.464	3.923			5.886	4.114	4.765	2.456	11.818	4.543	1.677	2.386	4.858
Gujarat	M	41.928	32.883	4.483	3.554	5.24	2.629	3.185		2.721	4.703	3.547	4.354	1.905	8.863	3.232		1.819	6.812

Gujarat	F	56.815	44.494	5.859	5.461	6.882	3.836	4.823		3.965	6.198	5.152	6.052		2.7654	12.574	4.901		1.989	2.553	4.520
Gujarat	M	46.41	36.031	4.951	3.97	6.45	2.646				5.732	3.635	4.77							2.106	5.400
Gujarat	M	47.418	37.367	4.984	4.003	6.532	2.796				5.717	3.644	4.568			10.263	3.943			2.135	6.357
Gujarat	M	51.152	40.038	5.525	3.128	6.096	3.115				5.399		5.094			10.883	3.974			2.268	6.516
Gujarat	M	50.941	40.242	5.214	4.422	6.657		3.103			5.886	3.865	4.954		1.802	10.702	4.078			2.493	5.666
Gujarat	F	49.183	37.987	4.952	3.915	5.783	2.961				5.555	4.069	4.81			10.909	4.346		1.605	2.109	4.852
Gujarat	F	37.233	29.699	4.503	4.688	5.08	2.386	3.485			4.65	3.521	3.796		1.343	7.99	3.41			1.742	4.848
Gujarat	M	40.064	31.87	4.664		4.96		2.391			4.375	3.522			1.857	8.544	3.35		1.291	1.762	3.781
Gujarat	F	37.939	30.258	4.744	3.68	5.41	2.361				4.446	3.345			1.705	7.625	3.039		1.159	1.75	5.315
Gujarat	F	44.764	43.9	4.501	3.828	5.579					4.832	3.962	4.706		1.806	9.609	3.356			2.004	4.584
Gujarat	M	36.247	28.33	3.962	3.191	5.19	2.322				4.591	3.418	3.772		1.466	7.801	3.153			1.784	4.617
Gujarat	F	35.522	28.18	3.758	3.259	4.57	1.663	3.189			4.015	3366	3.711		1.687	8.425	3.17		1.232	1.697	4.563
Gujarat	M	37.937	30.174	4.589	3.716	6.048	2.363	3.903		2.967	5.302	3.211	3.999		1.914	7.641	3.095			1.659	4.748
Gujarat	M	43.185	34.167	4.709	3.93	5.466	2.608	3.499			4.876	3.593	4.707		1.879	8.721	3.632			1.941	4.950
Gujarat	M	45.197	34.337	4.85	4.458	6.407	2.914				5.951	3.919	5.075		2	10.7	4.191		1.405	2.013	6.244
West Bengal	F	39.527	31.661	4.14	2.942	5.004	2.382	3.008			4.403	3.013	3.536		1.419	7.726	2.969			1.655	5.111
West Bengal	M	39.697	31.866	4.72	3.421	7.3	2.45	3.274			4.564	3.18	3.822		1.761	8.789	3.638			1.895	5.416
West Bengal	M	41.173	33.436	4.4	3.804	5.236	2.58	3.855			4.299	3.252	3.826		2.02	8.018	3.427			1.79	5.913
Sasson Dock	M	40.389	30.894	4.084	3.607	4.993	2.293	3.1	2.359		4.574	3.394	3.962	4.016	1.438	9.731	3.486	9.437	1.349	1.78	4.221
Sasson Dock	M	42.284	30.563	4.129	3.681	5.619	2.506					3.143								1.804	4.775
Sasson Dock	F	49.545	38.571	5.138	4.653	7.173	2.948	4.874			5.929	4.262	4.853	4.41	1.625	10.133	3.764	10.773		3.54	5.184
Sasson Dock	F	52.582	41.251	5.034		7.67	2.813	4.607	2.253	3.777	6.597	4.237	5.123	5.172	1.467	11.183	3.628	10.799		2.461	5.570
Sasson Dock	F	50.418	37.955	4.945	4.864	6.695	3.174	4.238		3.86	6.127	4.281	5.461		1.606	10.835	3.944	10.562	2.511	4.959	4.936
Sasson Dock	M	46.678	36.02	4.414	3.919	5.647	2.47	3.915	1.975		4.86	3.649	4.767	4.17	1.571	10.663	4.255	9.985		1.917	5.390
Sasson Dock	F	41.56	32.304	4.437	4.019	5.616	32.398	3.606		3.013	5.004	3.303	3.981	3.386	1.336	9.183	3.443	8.669		1.809	4.438
Sasson Dock	M	50.817	40.146	8.607	4.592	6.499	2.898	4.139			5.915	3.953	5.226		2.585	10.679	3.922	10.533		2.134	5.625
Sasson Dock	M	44.763	34.76	4.425	3.983	5.818	2.585				4.561	3.273			1.399	10.069	3.909	9.382		1.924	5.097
Sasson Dock	M	42.888	32.857	4.65	4.019	6.608	2.822	4.219		3.148	5.353	3.625	4.515	4.149	1.72	9.426	3.873	9.242		1.967	5.413

Sasson Dock	F	58.743	45.986	6.965	5.74	8.989	3.688	5.996		6.139	8.305	4.644	5.997	5.069	1.83	12.861	4.868	12.502	2.11	2.6	6.272
Sasson Dock	F	57.131	44.788	6.331	4.957	7.343	3.596	4.42		4.352	6.866	4.566	5.841	5.135	1.807	12.658	4.722	12.52		2.578	5.992
Sasson Dock	F	57.097	44.511	6.257	5.035	7.504	3.589	4.5277	2.73	4.737	6.943	4.452	6.103	5.343	2.003	13.232	4.833	13.185	1.848	2.516	6.419
Sasson Dock	F	52.595	41.542	6.398	5.274	8.562	3.456	6.113		4.574	7.58	4.401	5.209	4.791	1.716	11.459	4.041	11.146		2.349	5.580
Sasson Dock	F	55.734	43.604	6.452	5.585	8.596	3.354	6.357		4.104	7.429	4.605	5.701	5.741	2.015	10.957	4.1167	10.827		2.488	6.023
Sasson Dock	M	50.927	39.844	6.003	4.917	7.311	3.142	5.052			6.163	4.01	4.766	4.671	1.639	11.018	4.227	11.106		2.251	5.343
Sasson Dock	F	46.489	36.211	5.11	4.129	6.433	2.733	4.194			5.354	3.948	4.757	3.955	1.528	10.569	3.829	9.859		2.011	5.193
Sasson Dock	M	47.567	36.88	4.494	4.15	5.074	2.692				4.58	3.693	4.795	3.591	1.921	10.529	3.805	9.433	1.416	1.969	5.325
Sasson Dock	M	42.735	32.584	4.591	3.522	5.73	2.595	3.208			4.753	3.34	3.856		1.808	9.975	3.704	9.583	1.284	1.833	4.609
Sasson Dock	M	43.61	34.104	4.717	3.764	5.896	2.784	3.456		2.826	4.817	3.384	4.034	3.417	1.816	9.856	3.411	9.371	1.319	1.866	4.592
Sasson Dock	M	50.147	39.319	5.083	4.164	6.363	2.991				5.096	3.75	4.457		2.187	10.855	4.071	10.467	1.603	2.809	5.631
Sasson Dock	F	46.195	37.321	5.107	4.44	6.28	2.933	4.416	2.275	3.698	5.733	3.913	4.76	4.184	1.772	9.781	3.846	9.92	1.612	2.07	5.127
Sasson Dock	F	48.582	37.666	5.146	4.907	6.539	2.825	4.251		3.789	5.811	4.04	5.361	4.276	1.848	10.987	4.535	10.115		2.105	6.047
Sasson Dock	F	55.551	43.347	6.049	5.462	8.889	3.795	5.87		4.295	7.911	4.421	5.423	5.107	1.995	12.772	4.886	12.698		2.541	5.981
Sasson Dock	F	48.454	37.336	4.916	4.862	6.832	3.157				6.277	4.031	4.501	4.256	1.802	10.62	3.909	10.573		2.334	4.958
Sasson Dock	M	45.745	35.653	4.529	4.203	5.435	3.087	3.439		3.11	5.13	3.682	4.537	4.18	1.748	10.205	3.879	9.998		2.009	5.634
Sasson Dock	F	49.715	39.108	5.391	4.903	6.939	3.426	4.694		3.426	6.861	4.412	5.076	3.913	1.734	11.267	4.706	10.351		2.359	6.09
Sasson Dock	F	50.071	39.544	4.931	4.764	6.255	2.927	4.613		4.038	6.014	4.143	5.225	4.085	1.755	10.817	4.036	10.134		2.303	5.265
Sasson Dock	F	49.806	39.141	5.432	4.5	6.978	3.213	4.791			6.289	3.775	4.535	3.793	1.85	10.684	3.834	10.143	1.766	2.311	4.902
Sasson Dock	F	49.251	38.411	5.532	4.547	6.88	3.5				6.374	3.8	5.209	3.629	2.059	11.073	4.451	9.95		2.39	5.073
Sasson Dock	F	48.134	37.59	5.679	4.025	6.921	3.315	4.364	2.633	4.159	6.143	3.672	4.391	3.532	1.77	10.364	3.691	10.291	2.052	2.155	4.439
Sasson Dock	F	48.482	38.359	6	5.046	8.138	2.896				6.847	3.999	4.678	3.987	1.99	10.395	4.132	10.132		2.222	4.802
Sasson Dock	F	45.819	38.099	5.649	4.583	6.717	2.814	4.139			5.95	4.186	5.123	4.099	1.669	7.36	4.042	7.396		2.154	5.586
Sasson Dock	F	44.179	34.174	4.982		6.438	2.819				5.531	3.93			1.768	10.03	3.499	9.233		1899	4.909
Sasson Dock	F	54.311	42.505	5.951	5.219	8.055	3.364	5.036	2.647	4.88	7.283	4.21	5.364	4.817	1.911	12.413	4.524	12.53		2.534	5.594
Sasson Dock	F	53.907	43.09	5.768	5.355	8.421	3.587				7.556	4.387	5.462		1.843	11.311	4.502	11.313		2.45	5.636
Sasson Dock	F	52.829	41.775	5.338	4.6777	6.647	3.173				6.125	4.373	5.517	4.7784	2.045	10.93	4.281	10.46		2.313	5.871
Sasson Dock	F	52.859	41.238	5.698	5.267	8.798	3.189	6.355	2.528	4.922	7.836	4.311	5.62		1.855	11.609	4.46	11.255		2.282	5.563

Gujarat	F	56.441	44.902	6.117	5.051	8.837	3.422		7.779	4.383	4.752	4.58	2.361	14.609	5.108	13.65		2.543	5.522
Gujarat	F	45.495	35.449	5.359	4.228	6.319	2.992	4.566	5.446	3.781	4.272	4.055	1.8	10.274	3.895	9.769	1.481	1.892	5.167
Gujarat	F	49.408	37.785	5.675	4.575	7.235	3.545	4.461	6.455	4.111	5.069	44.269	1.946	11.365	3.999	11.183		2.398	5.662
Gujarat	F	47.855	37.38	4.913	4.977	7.367	3.243		6.574	4.072	5.073		1.933	11.078	3.842	11.56		2.303	5.652
Gujarat	F	44.672	34.644	5.494	4.277	6.96	3.257		6.509	3.581	4.257	3.96	1.903	10.397	3.684	9.798		2.188	4.547
Gujarat	F	50.27	38.369	5.386	4.825	6.797	3.477		6.502	3.89			2.115	11.117	3.605			2.378	5.073
Gujarat	F	55.132	42.114	6.86	5.262	8.645	4.017		7.515	4.709	5.278	4.826	2.46	12.067	4.399	11.527		2.529	6.035
Sasson Dock	F	47.401	36.796	4.775	4.096	6.398	2.861	4.054	5.464	3.808	5.091	3.959	2.048	9.827	3.695	8.906		1.969	5.147
Sasson Dock	F	43.113	33.358	5.767	4.03	6.911	2.92		5.03	3.733	4.124		2.043	10.497	3.848	9.329		2.029	4.759
Sasson Dock	F	43.288	33.893	5.767	4.285	6.55	3.331	4.257	6.034	3.498	4.145	3.855	1.535	9.501	3.162	9.8779	1.446	1.952	4.271
Sasson Dock	F	41.879	32.345	4.319	3.782	4.687	2.662	3.239	4.626	3.587	4.09	3.88	1.488	10.249	3.475	9.408	1.248	1.793	4.190
Sasson Dock	M	43.202	33.683	3.944		5.179	2.503	2.593	4.69	3.848	4.166		1.426	10.083	3.428	9.423		1.98	3.675
Sasson Dock	M	43.276	33.32	4.01	3.86	5.107	2.511	3.686	4.776	3.6	4.582	4.333	1.748	10.007	3.807	8.405	1.269	1.901	5.222
Sasson Dock	M	42.01	32.535	4.687	3.809	6.043	2.234	3.752	4.563	3.086	4.024	3.55	1.454	9.411	3.492	8.623		1.621	4.648
Sasson Dock	M	41.153	32.391	3.788	3.381	5.002	2.36	3.077	4.324	3.36	3.964	3.541	1.445	8.636	3.476	8.438		1.703	4.928
Sasson Dock	M	44.097	34.34	4.598	3.808	5.462	2.646		5.073	3.322	4.18	3.742	1.445	10.096	3.855	9.126		2.018	5.005
Sasson Dock	F	42.433	33.063	4.705	4.365	6.191	2.639	3.857	5.29	3.74	4.111	3.511	1.684	9.658	3.648	8.932		1.907	5.040
Sasson Dock	F	53.216	41.8	5.948	5.833	7.309	2.974	5.901	6.302	5.122	7.221	6.231	1.809	11.35	4.01	10.575	1.836	2.246	5.930
Sasson Dock	F	49.126	37.733	5.701	4.318	6.819	2.956	4.496	6.181	3.878	4.694	3.923	1.722	11.344	4.004	10.53		2.041	5.076
Sasson Dock	F	51.334	39.374	6.127	5.263	7.503	3.376	4.901	7.335	4.394	5.73	4.498	2.725	11.894	4.377	11.449		2.387	5.537
Sasson Dock	F	57.222	44.206	5.105	4.438	6.527	2.724	4.163	5.47	4.143	6.172	4.478	2.452	12.846	4.532	13.261		2.286	6.212
